# Transgender and gender diverse individuals embodying endometriosis: a systematic review

**DOI:** 10.3389/fmed.2024.1430154

**Published:** 2024-11-19

**Authors:** Maddalena Giacomozzi, Jip Bouwens, Stéphane Guy Aubin, Hester Pastoor, Petra Verdonk, Annemiek Nap

**Affiliations:** ^1^Department of Obstetrics and Gynaecology, Radboud University Medical Center, Nijmegen, Netherlands; ^2^Treat it Queer Foundation, Nijmegen, Netherlands; ^3^Erasmus University Rotterdam, Rotterdam, Netherlands; ^4^Department of Obstetrics and Gynaecology, Erasmus University Medical Center, Rotterdam, Netherlands; ^5^Department of Ethics, Law and Medical Humanities, Amsterdam University Medical Center, Amsterdam, Netherlands

**Keywords:** endometriosis, transgender, trans man, embodiment, pelvic pain, systematic review

## Abstract

**Background:**

Transgender and gender diverse (TGD) people embody social and health inequalities that disproportionately affect this community more than the cisgender population. Endometriosis is a chronic condition of the reproductive tract that affects 5–10% of cisgender women. A recent systematic review with meta-analysis uncovered a pooled prevalence of 25.14% among TGD individuals undergoing gender-affirming surgeries.

**Objective:**

This study aims to investigate the causes of the gap in prevalence of endometriosis between the TGD community and the cisgender population.

**Methods:**

A systematic review with a fit-for-framework analysis was conducted. Results were analysed according to the adjusted developmental framework for embodiment with an intersectional approach. Sources were categorised in multi-levels relating to the framework mechanisms of expression, shaping, interaction, and incorporation.

**Results:**

Four hundred twenty-three (423) studies published between 2001 and 2024 in English and Spanish were identified on the PubMed, Web of Science, Sociological abstracts, and PsycInfo databases. Thirty-two (32) peer-reviewed sources were selected.

**Discussion:**

The higher prevalence of endometriosis among TGD people compared to the cisgender population reflects a complex phenomenon whereby individual biomedical characteristics, and psychological and environmental factors interplay on multiple levels throughout one’s lifespan. The prevalence gap is striking in a context where TGD people experience great barriers and delays to access healthcare, and endometriosis is typically understood as a “women’s disease.” TGD people express lifestyle and environmental factors correlated with endometriosis more often than cisgender women, such as history of trauma, low self-image, obesity. Endometriosis interacts with one’s quality of life, and especially with gendered expectations related to menstruations, family planning and sexuality. This interference can result in biographical disruption and gender self-perception changes in both cisgender and TGD people. Exogenous testosterone use as gender-affirming therapy results in amenorrhea in 80% of cases. However, endometrium and follicular activities are still reported upon testosterone use suggesting endometriosis may be active. It is hypothesised that testosterone use could lead to a hyper-estrogenic state that would stimulate endometriosis proliferation.

## 1 Introduction

### 1.1 Background

Physiological phenomena such as menstruation and reproduction are experienced differently by every human given the idiosyncrasy of physical characteristics, psychological features, and environment they live in (1). Health and disease manifest in one’s body as a result of a multi-level interplay of individual and societal factors (2). In other words, these are embodied in one individual experience (2). Furthermore, gender as a structure is embodied. The pervasive social understanding that bodies are, and should be, gendered, and that these are either female or male, is challenged by the gender dysphoria reported by a rapidly growing community of people, between the gender assigned to them by the environment and their self-perceived gender (3, 4). This literature review with systematic search and fit-for-framework analysis focuses on endometriosis, a disease closely related to menstruation and reproduction. It investigates how it is embodied among people with gender dysphoria and why it is more prevalent among TGD people than cisgender individuals.

Transgender and gender diverse (TGD) people are persons whose gender identity is incongruent with the gender assigned to them at birth (5). Transmasculine people are individuals assigned female at birth (AFAB), though they do not identify as women. They may identify—in binary terms—as transgender men. Alternatively, their gender identities may be positioned beyond the binary of woman/man. For example, they could be genderqueer, genderfluid, non-binary, or two-spirit. Gender identities and gender expressions are culturally and socially determined (6). They vary among different peoples and throughout historical trajectories. In the last decades, an increasing proportion of the general population in the Global North has disclosed TGD identities (3, 4, 7). Upward temporal trends for this population have been observed, especially for youth (4). Consequently, the number of TGD people of reproductive age, and thus potentially living with endometriosis, is expected to increase in the coming years (4).

TGD individuals have specific health needs which encompass, but are not limited to, gender-affirming care (GAC) (5, 8). Some TGD people seek professional help for gender affirmation. GAC is a multidisciplinary field that sees the involvement of different disciplines including medicine, psychology, speech therapy, and sexology (5). GAC is presently still centred on endocrinological and surgical treatments. Transmasculine individuals make common use of exogenous testosterone as masculinizing hormone therapy. Testosterone is administered transdermally or intramuscularly (5, 8). In addition, or as an alternative to hormone therapy, transmasculine individuals may need gender-affirming surgeries such as mastectomies, hysterectomies, or oophorectomies. Beyond GAC, TGD people present with unique needs in relation to their reproductive health (8). In fact, transmasculine individuals experience chronic pelvic pain—defined as sexual or non-sexual related pain in the pelvic area present for longer than three months—more often than cisgender women (9–11). The underlying causes for this gap in prevalence have hardly been researched. This gap may relate to the gender-affirming therapies that transmasculine individuals use as well as their distinctive psychological and social background (10). Similarly, the prevalence of specific diseases related to chronic pelvic pain conditions among this population remains largely under-investigated (9, 11).

Endometriosis is a disease characterised by chronic and cyclic pelvic pain. It is a chronic disease defined by the presence of functioning endometrial-like tissue outside the uterus (12, 13), with its leading symptoms being dysmenorrhea (pain during menstruation), dyspareunia (painful intercourse), dyschezia (painful defecation), and dysuria (painful urination) (12, 14). Endometriosis typically presents with cyclic pain related to menstruations. However, in the long-term pain can become chronic and lead to sensitization and an overactive pelvic floor (15, 16).

Endometriosis is an oestrogen-dependent disease. Its treatment is based on hormonal suppression of the menstrual cycle and surgical excision of endometriosis lesions. Usually, the goal of hormonal therapy is suppression of the menstrual cycle that results in secondary amenorrhea (12, 17). First choice in hormone treatment usually consists of progestins-only or continuous combined-oral contraceptives (12). Second choice medications are GnRH-analogues (12). When conservative treatment fails, a surgical approach is considered to remove endometriosis lesions (14). The surgical procedure and techniques involved are highly dependable on the location and size of the lesions. Some examples of routinely performed surgery for endometriosis treatment are total laparoscopic hysterectomy, cystectomy, and, more occasionally, oophorectomy. To note: the same surgeries are sometimes performed as gender-affirming surgeries for transmasculine people (5).

The quality of life of people living with endometriosis is significantly affected by this condition (13, 18–20). The chronic nature of the symptoms often leads to the disruption of one or more life domains (18, 21). Complaints are reported to frequently interfere with the social life, intimate and sexual relationships, family planning, and professional ambitions of the patient and their partners/family (21). The consequences of endometriosis are multidimensional and can be life-long (14, 18).

Endometriosis can affect any person with a uterus and ovaries, but very few studies have focused on the TGD population affected by this disease (22). A recent systematic review highlighted a higher prevalence of endometriosis in TGD patients than in the cisgender population (23). The pooled prevalence of endometriosis in this population was calculated to be 25.14% (17.24–33.94%), compared to 5–10% in the general population (23). The incidence of TGD people presenting with dysmenorrhea during testosterone use without other medications was 70.58% (63.87–80.91%) (23). A study among TGD youth reported persistent dysmenorrhea in 33% of the cases after initiation of testosterone use (24). The same study found that endometriosis was confirmed in all the patients who underwent laparoscopy for gender-affirming surgeries (24). Ferrando et al. (25) observed that people using testosterone who presented with breakthrough bleeding, lack of secondary amenorrhea, and heavy menstruations were more at risk of endometriosis than amenorrheic patients using testosterone.

The high prevalence of endometriosis among TGD people makes it imperative to research the underlying causes for the gap in prevalence between the TGD and the general population. Endometriosis can significantly impair quality of life, as it impacts various domains including sexual and mental health. This burden is particularly pronounced among transgender and gender-diverse (TGD) individuals, who may present an increased vulnerability compared to cisgender individuals (26, 27), as they are subjected to additional stressors owing to the marginalisation of their identities and experiences. Given the disadvantaged social space that the TGD community holds and the multiple, severe barriers that this community faces in their access to adequate healthcare services, TGD-centred research is necessary to outline the social and health inequalities that disproportionately affect TGD people. Indeed, in addition to extensive delays in access to care or diagnosis, healthcare providers and institutions often do not offer competent services tailored to TGD-specific health needs. These particular barriers relate, for example, to the pervasive lack of education of healthcare professionals (HCPs) regarding gender diversity, as well as patient-driven delays due to gender dysphoria triggered by gynaecological examinations (27). Additionally, within the dominant cisheteronormative paradigm, endometriosis is narrated and framed as a “women’s disease” which reflects itself in the structural exclusion of TGD people from endometriosis care (27). The unique positioning of TGD people with endometriosis requires a deeper investigation of how they embody this condition to ultimately provide more accessible and adequate care for this underserved community.

### 1.2 Theoretical background

To explore the complexity of the gap in endometriosis prevalence between TGD and cisgender populations, a developmental perspective on embodiment (28) following Krieger’s (2) embodiment scholarship has been adopted for this epidemiological review. Embodiment is at the same time a construct and a process, and it flows from an eco-social matrix. The central premise of embodiment is that humans are simultaneously social beings and biological organisms (2). According to embodiment theory, any living organism embodies, i.e., literally incorporates, the social and ecological circumstances in which they are born, develop, and live. Bodies are seen as active and involved entities that interact in a multileveled ecosystem that has stratified over time. In the embodiment process, living beings are shaped by their engagement with the environment. Their shaping is determined by the historical and ecological contexts which result in specific biological characteristics (2, 28). Embodiment theory aims to explain differences in biological characteristics including unequal distribution of a disease. For this, societal contexts are investigated to explore how they could result in different expressions of bodily characteristics. Adopting an embodiment theoretical approach has the purpose to support conducting research with rigorous scientific methodologies and to promote social and health equity (2, 28).

For example, literature has shown that individuals from lower socioeconomic status (SES) often face a higher exposure to air pollution (29). Women living in poorer neighbourhoods are additionally more exposed to indoor and outdoor air pollution due to various binary gendered roles and activities, such as cooking for many hours a day (30). Air pollution in turn has been linked to numerous adverse health effects, including respiratory diseases like lung cancer (31). Using embodiment theory encourages us to consider the broader social context in understanding the relationship between health outcomes and social inequalities.

Adopting a developmental perspective on embodiment allows us to consider the temporalities that are part of the process (28, 32, 33). This is particularly helpful when researching chronic conditions such as endometriosis that develop throughout different stages of life, in combination with gender, which is itself a social construct that tends to change significantly over time. This approach offers the analytical tools to examine the interconnectedness of the different levels as they relate to one another over a lifespan. Such a framework is intrinsically interdisciplinary—nature expands the explanatory capacity of the biomedical paradigm alone that correlates single determinants and mechanisms with outcomes decontextualizing and disembodying the phenomena. An adjusted version of the developmental perspective based on the embodiment framework (28) has been selected for investigating the epidemiological inquiry of this study because it provides the theoretical approach necessary to analyse the multileveled biomedical as well as psycho-social mechanisms underlying the phenomenon of endometriosis among TGD people throughout its lifespan. TGD individuals embody unique intersections of biomedical characteristics related, but not limited to, gender-affirming care, psychological features including gender dysphoria and social marginalisation, and minority stress. Endometriosis interferes with one’s quality of life at different timepoints associated with puberty, sexual development, and family planning. These life processes are experienced differently by TGD and cisgender people and a developmental embodiment approach to endometriosis enables an understanding of how these may differ in these two social groups when affected by this condition.

**Figure 1:**
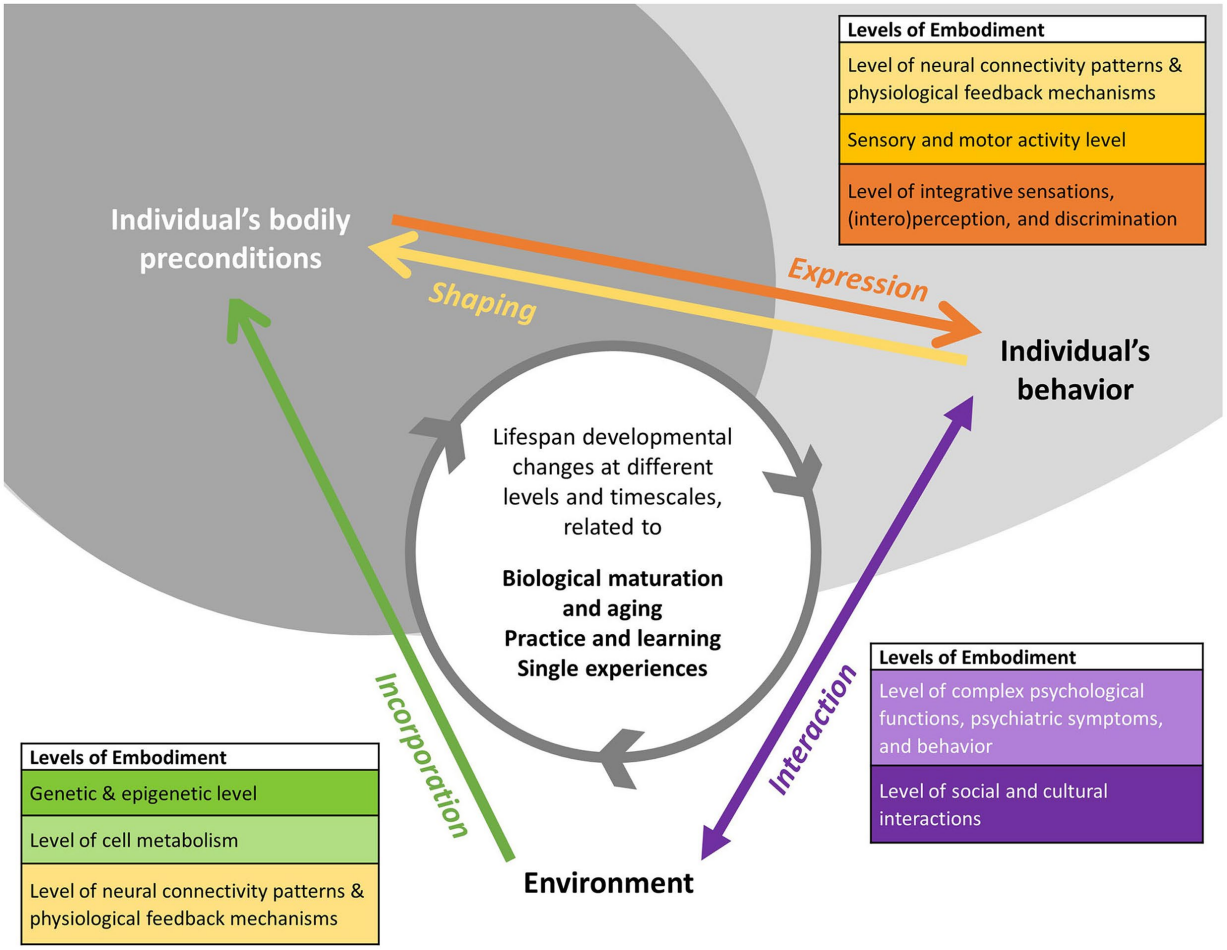
Developmental perspective based on embodiment framework (Reprinted with permission from Lux et al. (28), licensed under CC BY).

In this framework four embodiment processes (indicated by arrows) are examined (Figure 1) (28). These are: expression, shaping, interaction, and incorporation. Each process starts in a domain and indicates a certain analytical perspective by pointing towards another domain. For example, the green arrow originating in the environment domain and pointing at individual bodily preconditions signals an environmental approach that analyses the incorporation of experiences into bodily characteristics. There are three domains: an individual’s bodily preconditions, an individual’s behaviour, and environment. Each domain has multiple interconnected levels as represented in the rectangles. The lifespan developmental changes processed are indicated by the grey circular arrow.

**Figure 2:**
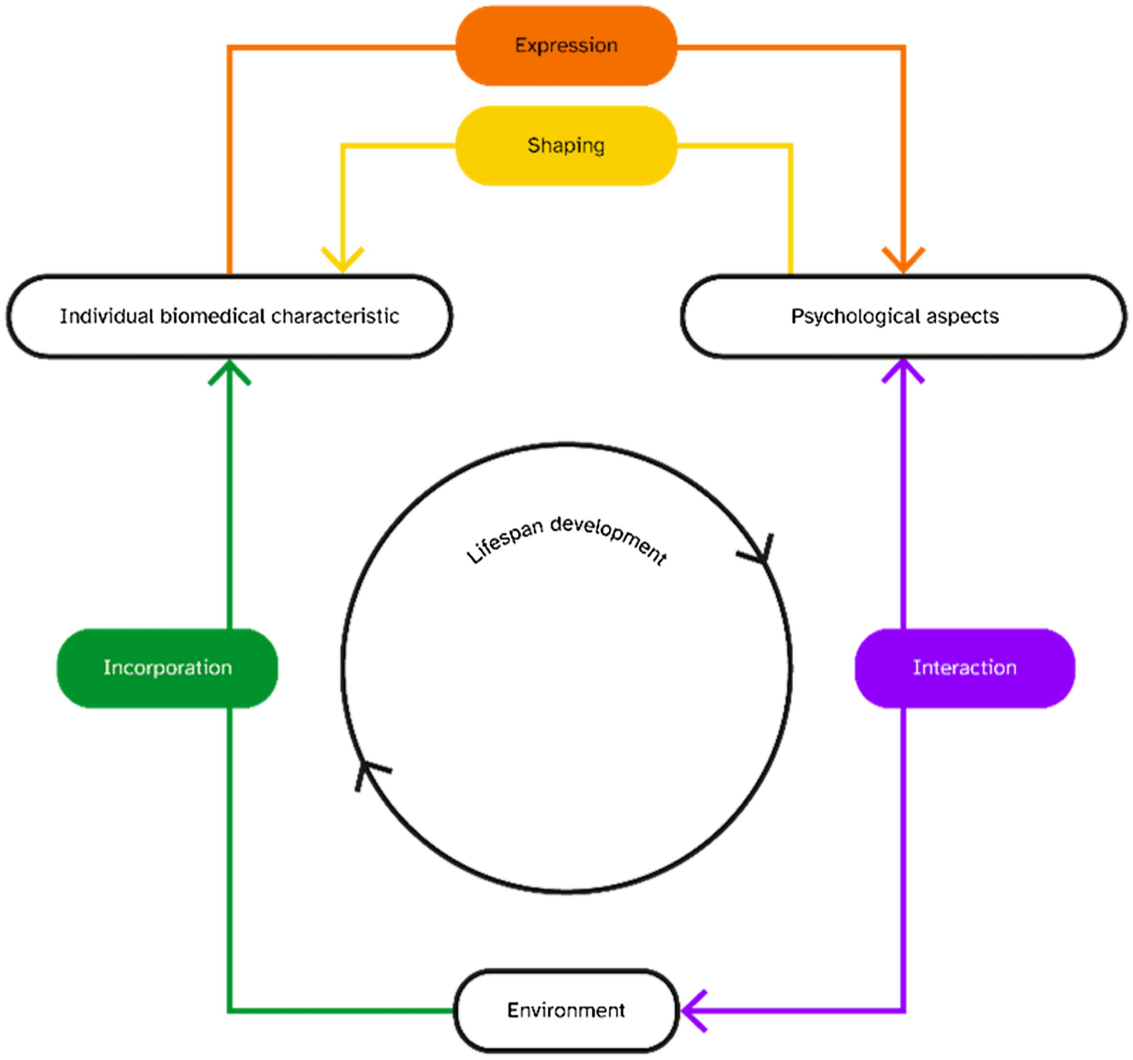
Adjusted developmental perspective based on embodiment framework.

The adjusted version of this framework (Figure 2) implies three main changes compared to the original framework. Firstly, it focuses on individual biomedical characteristics instead of bodily preconditions because the physical characteristics that TGD people embody throughout their lifespan tend to change significantly with gender-affirming therapies. They are thus not preconditions but rather characteristics *in itinere* that keep changing throughout one’s life. Secondly, the framework explores psychological aspects at large rather than being restricted to individual behaviour so as to include personal feelings, attitudes, and other psychological features relevant to this review such as gender dysphoria. Thirdly, the levels of embodiment have been simplified into single domains to increase accessibility and comprehensibility of the theoretical framework as it has been applied specifically to this study.

## 2 Materials and methods

A systematic search with a fit-for-framework analysis was conducted to answer the research question. This study was exempt from Ethics Committee Review due to its selected methodology, and it was not registered on PROSPERO Studies published between 2001 and 2024 in English, Dutch, Italian, or Spanish were identified. The search was conducted on four databases: PubMed, Web of Science, Sociological Abstracts, and PsycInfo. A sample of search terms used was sexual and gender minorities; transsexualism; gender identity; endometriosis; dysmenorrhea.

There was no restriction in study design. Studies on AFAB individuals, including not only cisgender women and transgender men, but also gender diverse people across a wide spectrum of gender identities, with confirmed endometriosis or endometriosis symptoms, were included. Endometriosis symptoms that were searched included chronic abdominal/pelvic pain, heavy menstrual bleeding, dysmenorrhea, dyspareunia, dyschezia, and dysuria. Therefore, studies discussing (one or multiples of) these symptoms were included even if they were not focusing on endometriosis. Studies including endometriosis patients’ partners were also selected. Outcome measures could include any information describing the experiences of a person living with endometriosis as well as clinical parameters related to individual biomedical characteristics or psychological aspects of these persons.

Studies that were not focusing on our desired outcome measures were excluded. Studies were excluded when pelvic pain symptoms were caused by other conditions than endometriosis.

Screening of the retrieved articles was done using Rayyan, a research collaboration platform facilitating blinded reviews with more than one author. First, duplicates were removed. After that, articles were screened on title and abstract by two reviewers. Reviewers were blinded from the other reviewers’ decisions until every title and abstract was screened. Any conflicts were resolved by discussion with a third reviewer until an agreement was reached. Screening for full-text eligibility was done in the same manner. With the reference lists of the included articles, the backwards snowballing method was used to identify any other relevant articles.

Data extracted from each study included: first author, publication year, country of origin, study type, purpose, study population, sample size, outcome variables, and findings. The quality of individual articles was assessed using the LEGEND (Let Evidence Guide Every New Decision) appraisal forms developed by the James M Anderson Center for Health Systems Excellence at Cincinnati Children’s Hospital Medical Centre. The quality assessment used a scale ranging from 1 to 5, with 1 indicating the highest quality level and 5 the lowest. Additionally, each level was subdivided in ‘a’ indicating good quality or ‘b’ representing lesser quality.

Data was extracted separately by two reviewers and the relevance for and place within the different mechanisms of our chosen framework was subsequently discussed. This was done by clustering key findings into the different embodiment processes, i.e., expression, shaping, interaction, and incorporation.

When an outcome described how individual biomedical characteristics are embodied and expressed in an individual’s psychological aspects like their behaviour, personal feelings, and attitudes, it was labelled as expression. If the source described how psychological aspects shape biomedical characteristics, it was labelled as shaping. Findings describing the interplay between a person’s psychological aspects and their environment were labelled interaction. Data illustrating the embodiment of experiences at different biological levels in the interaction with one’s environment were labelled as incorporation.

## 3 Results

**Figure 3:**
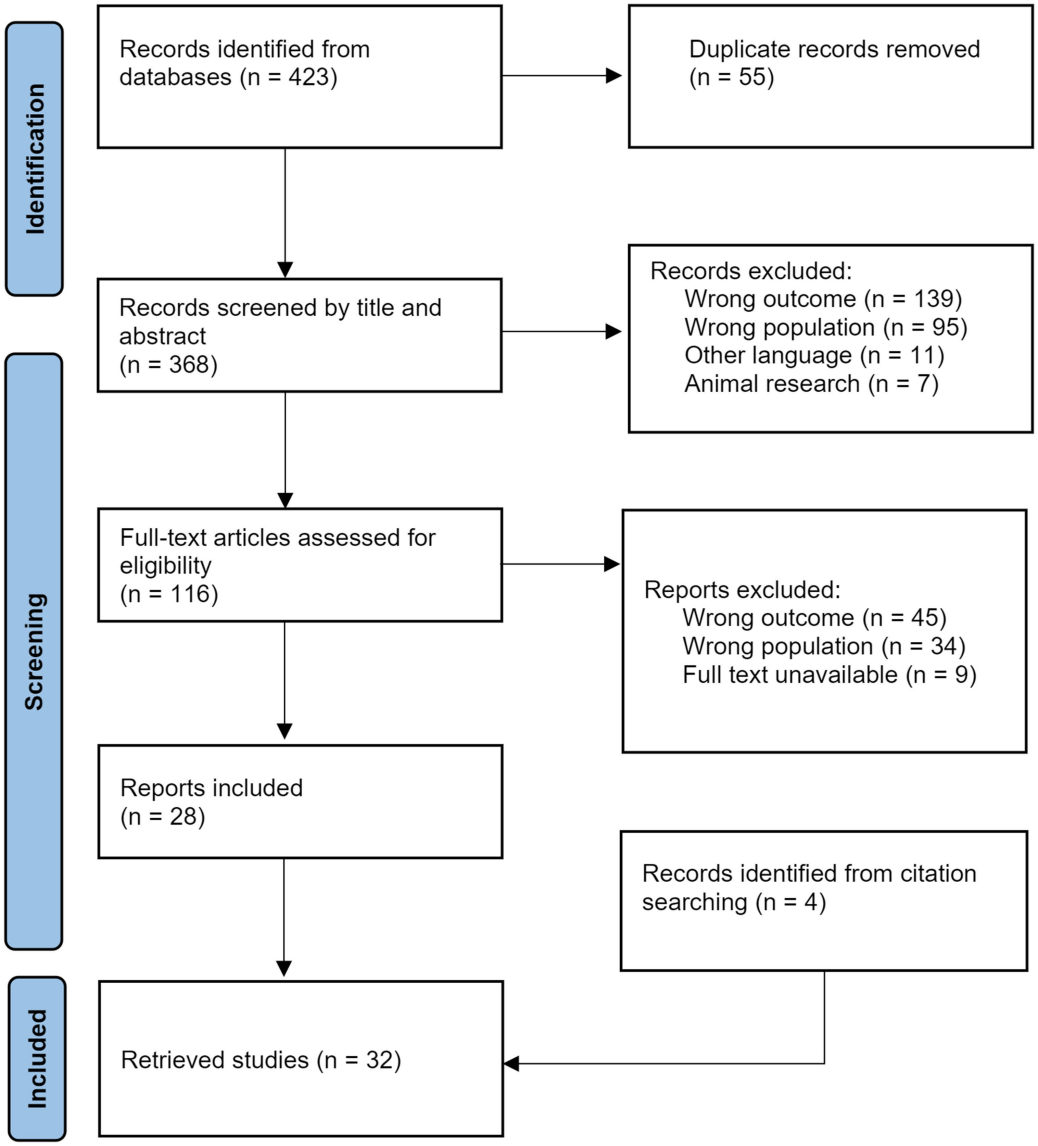
PRISMA flow chart illustrating the number of records included and excluded at various screening and reviewing steps, leading to a final list of records for data extraction.

The initial search found 423 peer-reviewed articles (Figure 3). After duplicates (n = 55) were removed, 368 sources were screened by title and abstract. Of these, 252 were excluded—139 due to wrong outcome, 85 due to wrong population, 11 due to other languages and 7 because they focused on animal research. Full-text eligibility was performed on 116 full-text articles and subsequently 88 sources were excluded—45 due to wrong outcome, 34 due to wrong population, and 9 because no full-text was available. Eventually, 28 articles were retrieved and their references crossed-checked. Citation-searching led to the addition of 4 sources, resulting in the final set of 32 included articles.

The included articles have been presented in Table 1. For each article were indicated: year of publication, author, country of origin, methodology, evidence level, purpose, population and sample size, data collection, outcome measures, and key findings.

**Table 1:** Summary and metadata of included articles.

Author(s) + year of publication	Country of origin	Language	Method	Final evidence level	Purpose	Population + sample size	Data collection	Outcome measures	Key findings
Jones (2021) (22)	USA	English	Qualitative	2b	Examine where queerness meets gendered ableism	Cisgender and queer people with endometriosis (69)	Literature review	Recommendations for more inclusive and accurate language and practices in endometriosis care	Endo discourses must centre queer people and practices and disability to yield better outcomes because the experiences of those marginalised in endo discourses are crucial to better care for all.
Shim et al. (2020) (24)	USA	English	Quantitative	4a/4b	Study the presentation of dysmenorrhea and endometriosis in transmasculine adolescents and review their treatment outcomes	Transmasculine persons <26 years old (35)	Retrospective review of clinical records	Clinical characteristics, transition-related care, and treatment outcomes	Evaluation of endometriosis was underutilised in transmasculine persons with dysmenorrhea. There is individual variation in the susceptibility to menstrual and pain suppression by androgens, danazol and testosterone.
Ferrando et al. (2021) (25)	USA	English	Quantitative	4a	Describe incidence of pelvic pain in transgender men undergoing hysterectomy for gender affirmation and describe the incidence of endometriosis found at the time of surgery	Transgender men undergoing hysterectomy for gender affirmation (67)	Retrospective cohort study with data obtained from the patients records, operative reports and pathologic reports	Pelvic pain, intraoperative endometriosis	50.7% (95% CI 39.7–63.2%) of patients complained of pelvic pain, intraoperative endometriosis was diagnosed in 26.9% (95% CI 18.0–39.0) of patients, use of testosterone was not associated with the presence of endometriosis.
Brown and Weikel (2022) (34)	USA	English	Qualitative	5b	Educate providers on how to identify, workup, diagnose, and treat common causes of non obstetric gynecologic pelvic pain	Persons with non obstetric gynecologic pelvic pain (25)	Literature review	NA	Risk factors for primary dysmenorrhea are: age younger than 30 years, menarche before 12 years old, obesity or body mass index less than 20 kg/m^2^, cigarette smoking, heavy and/or long duration of menstrual flow, history of sexual assault, family history of dysmenorrhea. There are descriptions of genetic components to development of endometriosis.
Cao et al. (2021) (35)	USA	English	Quantitative	4a	Describe clinical characteristics of transmasculine individuals who underwent hysterectomy + characterise surgical pathology findings	Transmasculine individuals who underwent a hysterectomy (72)	Retrospective identification by examination of patient files for markers of transmasculine identity	testosterone use, comorbidities, surgery indication, pathology findings	Pelvic pain was the leading indication for surgery (90%) followed by gender dysphoria (26%). 58% of patients reported a history of a mental health condition, with 47% of patients using antidepressants or mood stabilisers.
Grimstad et al. (2021) (36)	USA	English	Quantitative	4a	Evaluate breakthrough bleeding in TGD (young)adults on testosterone therapy for gender affirmation	TGD AYA on T-GAHT >1 year (232)	Retrospective cohort study with data obtained from the patients records	Presence of, and risk factors for, breakthrough bleeding	Patients with breakthrough bleedings were on testosterone therapy longer and were more likely to have endometriosis.
Grimstad et al. (2019) (37)	USA	English	Quantitative	4a	Describe characteristics of uterine pathology after initiation of testosterone in transmasculine persons	Transgender persons on testosterone therapy undergoing hysterectomy for gender affirmation (94)	Retrospective cohort study with data obtained from the patients records, operative reports and pathologic reports	Characteristics of uterine pathology	Endometrial activity persisted, despite the finding that the majority of patients had amenorrhea.
Grimstad et al. (2020) (38)	USA	English	Quantitative	4a	Describe characteristics of ovarian histopathology after initiation of testosterone in transmasculine persons	Transgender persons on testosterone therapy undergoing oophorectomy + hysterectomy for gender affirmation (85)	Retrospective cohort study with data obtained from the patients records, operative reports and pathologic reports	Characteristics of ovarian histopathology	Pathologic descriptions suggest both ongoing physiologic activity and no evidence of increased malignant pathology despite long term exogenous testosterone exposure.
Kaltsas et al. (2024) (40)	Greece	English	Qualitative	5a	Narrative review to identify gaps in research and care for transgender men with endometriosis	Peer-reviewed articles (81)	Narrative review	Gaps in research and clinical practice	Endometriosis in transgender men remains under-studied. Interaction between GAC and endometriosis treatment is poorly understood.
Moulder et al. (2020) (39)	USA	English	Qualitative	5a/5b	Describe the multifactorial nature of pelvic pain in transgender men	Transgender men (75)	Literature review	Secondary effects of systemic testosterone therapy on pelvic pain	Chronic pelvic pain is not uncommon in FtM patients, and may have a multifactorial etiology. It is important to consider systemic testosterone therapy and its effect on the reproductive system. Hysterectomy often appears therapeutic as both a gender-affirming procedure and pain treatment.
Ferrando (2022) (41)	USA	English	Qualitative	5a	Clarify existing knowledge gaps	Transmasculine individuals (8)	MEDLINE search	Knowledge gaps in literature	Transmasculine individuals can have a wish for hysterectomy for gender affirmations and/or because of pain symptoms. Traditional options for pain management may not always be appropriate in this patient group.
Ricci et al. (2016) (47)	Italy	English	Quantitative	1b	Establish whether physical activity influences endometriosis risk	Persons with endometriosis and controls (7520)	Literature review	Endometriosis risk	Physical activity may reduce the risk of endometriosis.
Facchin et al. (2017) (63)	Italy	English	Quantitative	4a	Determine psychological impact of putative predictors in women with endometriosis	Endometriosis patients (210)	Structured interviews, questionnaires (HADS, RRS)	(Individual differences in) mental health status (PROM)	Endometriosis is characterised by a remarkable variability in terms of symptoms, types of lesions, psychological and relational outcomes.
Rydström (2020) (42)	Sweden	English	Qualitative	5b	Theoretical intervention in the cisgendering of menstruation	Swedish trans people (9)	Interviews	Experiences of transgender persons who menstruate	Degendering menstruation is necessary to deal with menstrual shame, taboos, and stigmas in a gender-inclusive manner.
Chrisler et al. (2016) (50)	USA	English	Qualitative	2a	Gather information about attitudes toward and experiences with menstruation in the masculine of centre and transgender community	Masculine of centre and transgender people (150)	Questionnaires (BATM, ATMS) and additional (open ended) questions	Attitudes toward menstrual suppression	Attitudes to menstruation of masculine of centre and transmasculine persons are similar to those of cisgender women. Their attitudes towards menstrual suppression seem to be more positive.
Carvalho et al. (2024) (26)	Portugal	English	Qualitative	4a	Explore the extent of pain symptoms, sexual distress, mental health, and health profile of TGD people with endometriosis	Transmasculine and non-binary people with endometriosis (6)	Observational	Psychosocial and sexual variables	TGD people with endometriosis may experience worse mental health challenges, more constraints in sexual function, diminished experiences of pleasure, and augmented sexual distress compared to cisgender people.
Lutgendorf (2019) (51)	USA	English	Qualitative	5a/5b	Describe the effects of intimate partner violence (IPV) on women’s health	People experiencing IPV (69)	Literature review	Effect of Intimate Partner Violence on reproductive health	Intimate partner violence is an important problem in women’s health, in which obstetrician-gynaecologists have the knowledge and the tools to make a difference.
Nash (2023) (45)	Australia	English	Qualitative	2a/2b	Examine the additional labour involved in managing menstruation during remote Antarctic fieldwork	Women doing remote Antarctic fieldwork (50)	Interviews	Experiences of managing menstruation in male-dominated field teams, menstruation as taboo, strategies for managing menstruation in the field	In male-dominated spaces, expeditioners must go to great lengths to make their menstruation invisible, for which menstrual suppression technologies are a primary way to do that. The lack of infrastructure to support menstrual health in the field is a form of sexism that maintains women’s lower status in polar field environments.
Eder and Roomaney (2024) (56)	South Africa	English	Qualitative	3a	Report on transgender and non-binary people’s experience of living with endometriosis	Transgender and/ non-binary people with (suspected) endometriosis (11)	Semi-structured interviews and diaries during three months	Real lives experiences of transgender and non-binary people living with endometriosis	Transgender and non-binary people with endometriosis experience endometriosis symptoms that create an unhomelikeness with the body and their gender identity.
Ellis et al. (2024) (57)	Aotearoa New Zealand	English	Qualitative	3a	To assess the experiences of endometriosis patients who identify as LGBTQIA+in Aotearoa New Zealand	LGBTQIA+ people with endometriosis (28)	Only survey	Real lived experiences of LGTQIA+ individuals with endometriosis in Aotearoa New Zealand	LGBTQIA+ individuals with endometriosis in New Zealand have negative experiences, particularly associated with a focus of care on penetration, pregnancy, and in some cases, feminization.
Riazi et al. (2014) (52)	Iran	English	Qualitative	2a/2b	Explore the perception and experiences of endometriosis patients and physicians about occurrence and diagnosis of endometriosis	Endometriosis patients (12), gynaecologists (6)	Semi-structured interviews	Experiences of endometriosis patients and gynaecologists regarding occurrence and diagnosis of endometriosis	Perception of occurrence and diagnosis of endometriosis is a complex issue. Both patients and physicians indicated that they were seeking reliable diagnostic indicators.
Young et al. (2019) (53)	Australia	English	Qualitative	2a	Examine the language used by clinicians to construct medicine and women with endometriosis and identify whether these constructions endorse or challenged historical discourses surrounding endometriosis	Gynaecologists (8) and GPs (4)	Semi-structured interviews	Clinicians narratives and views on endometriosis	Clinicians endorsed medicine as the authoritative knowledge on women and their bodies and constructed it as being about providing answers on, and doing things to, the body. Women with endometriosis were constructed as reproductive bodies with hysterical tendencies, with hysteria most often being endorsed when discussing women for whom treatment was not helpful or who held a perception of their disease alternative to their clinician.
Yang et al. (2023) (54)	China	English	Qualitative	2b	Examine the gender ideologies behind male discourses about dysmenorrhea	Young Chinese males (26)	Search on Zhihu for questions and comments by male “netizens” about menstrual discourses	Male discourses and views about dysmenorrhea on social media	Chinese men have to some extent broken the traditional menstrual taboo, driven by the contemporary gender norms that stress men’s responsibility for their partner. Their support can alleviate the pain and appease negative emotions of their menstrual partner, but it is too soon to leap to the conclusion that Chinese women are treated with sufficient respect and equality in an intimate relationship.
Sang et al. (2021) (55)	UK	English	Qualitative	2a	Explain the relationship between menstruation and associated gynaecological health conditions and employment for women and trans/non-binary people by introducing the concept of ‘blood work’	People working in higher education (627)	Survey	Experiences of menstruation, menopause, and gynaecological health conditions at work of persons working in higher education	Employees’ blood work comprises distinct difficulties that are related to the management of painful, leaking bodies, access to facilities, stigma, and balancing workload.
Cole et al. (2021) (58)	UK	English	Qualitative	2a/2b	Examine how women negotiate changes to their identity and relationships as a result of endometriosis	(Presumed) cisgender women (34)	Qualitative analysis of online survey responses	Experiences of women negotiating changes to identity while living with endometriosis	Women diagnosed with endometriosis may experience many barriers to reappraising their identity and relationships in the context of physical symptoms and psychological impacts of the condition.
Hudson et al. (2016) (59)	UK	English	Qualitative	2a	Explore the concept of biographical disruption in patients with endometriosis from a dyadic perspective	Couples of which one of the partners had endometriosis (22)	Semi-structured interviews	PROM	Endometriosis can result in disruptions, appraisals and revisions specifically in relation to sex and intimacy, planning for and having children, working lives and social lives.
Wright (2019) (44)	USA	English	Qualitative	2b	Reflect how biomedical and gendered perceptions of reproductive health can impact an illness experience	Author (1)	Essay	Author’s experience of endometriosis	Social scripts assigned to how women cope with menstrual pain can be rewritten through strong patient self-advocacy and knowledge.
Facchin et al. (2018) (48)	Italy	English	Qualitative	2a	Develop a grounded theory of how endometriosis affects psychological health	Endometriosis patients (74)	Open interviews, questionnaire (HADS)	PROM	Experiencing disruption, as opposed to restoring continuity, involved higher distress and was associated with a long pathway to diagnosis, bad doctor-patient relationships, poor physical health, lack of support, negative sense of female identity, and identification of life with endometriosis.
Ariza-Ruiz e al. (2017) (43)	Colombia	Spanish	Mixed methods	2b	To investigate challenges and experiences related to menstruation in rural Colombia	Children and adolescents; formal and informal caregivers (246)	Mixed qualitative and quantitative research incl. Focus groups, in-depth interviews and field observations	Experiences of menstruation	The social and cultural constructs around menstruation were characterised negatively. This reinforced taboos resulting in gender-based discrimination, stigma and inequalities.
Jeffrey et al. (2024) (60)	Australia	English	Qualitative	5a	Identify barriers to accessing gender-affirming healthcare specific to gynaecology,	AFAB TGD individuals with endometriosis (70, 1)	Narrative review (70) and autoethnography (1)	Barriers to healthcare	TGD people with endometriosis or CPP currently have limited access to affirming medical care which results in poorer health outcomes.
Vallée, et al. (2023) (27)	France	English	Qualitative	5b	Raise awareness about endometriosis in transgender men,	Transgender men with endometriosis (31)	Narrative review	Needs for research, education and care for TGD people with endometriosis	Inclusive research, education and care are necessary to break down the barriers that hinder effective diagnosis and management of endometriosis in this marginalised population.
Ingudomnukul et al. (2007) (61)	UK	English	Quantitative	4b	Test whether women with ASC or mothers of children with ASC have an increased rate of testosterone-related medical conditions	Women with ASC (54), mothers of children with ASC (74), mothers of typically developing children (183)	Questionnaires (TMQ)	PROM	There is an association between ASC, “tomboyism,” bisexuality/asexuality, and unusually painful periods in adulthood.

NA, Not Applicable; T-GAHT, Testosterone for Gender Affirming Hormone Therapy; AYA, Adolescents and Young Adults; FtM, Female-to-Male; PROM, Patient-Reported Outcome Measure; TGD, Transgender and Gender Diverse; HADS, Hospital Anxiety and Depression Scale; RRS, Rumination Response Scale; BATM, Beliefs About Menstruation; ATMS, Attitudes Towards Menstrual Suppression; GP, General Practitioner; ASC, Autism Spectrum Conditions; TMQ, Testosterone-related Medical Questionnaire.

### 3.1 Analysis of the results

The results were analysed according to the adjusted version of the developmental perspective on the embodiment framework (28). Each article was examined to identify the outlined processes that could explain the difference in prevalence of endometriosis between the TGD and the general population. Processes were clustered corresponding to the framework, i.e., as expression, shaping, interaction, and incorporation. At least one possible underlying cause was identified for every process as outlined in the framework. The most frequently investigated processes were expression and interaction, followed by shaping.

### 3.2 Expression

The process of expression (orange arrow) indicates how an individual’s biomedical characteristics relate to one’s psychological features (Figure 2). TGD people may or may not have the same anatomical and physiological characteristics as cisgender women, or they may experience them differently. Gender-affirming care such as testosterone use and hysterectomies alter biomedical characteristics, for instance by interfering with the menstrual cycle. Biomedical characteristics of TGD people using testosterone that could be related to endometriosis, such as oestrogen levels and gonadal activity, will here be defined first, followed by descriptions of how these may be related to individual psychological features.

#### 3.2.1 Biomedical characteristics of TGD people using testosterone

Certain biomedical characteristics such as age of menarche are known risk factors for endometriosis. The retrieved studies did not investigate their prevalence among TGD people compared to cisgender women. Some authors reported baseline characteristics of the included TGD population, and these seemed not to vary from the known data in the general population, however no accurate comparative analysis was conducted between this group and cisgender women (34). No literature was identified about endometriosis-related somatic comorbidities in TGD patients.

In contrast, several studies assessed how anatomical and physiological changes following exogenous testosterone use could expose pathways to endometriosis onset (25, 35–39). These studies were conducted in patients undergoing hysterectomies or other gender-affirming surgeries. A retrospective study on pelvic pain among transmasculine patients undergoing hysterectomy (n = 67) found that 50.7% (n = 34) of the participants experienced pelvic pain before surgery (25). Endometriosis was diagnosed intraoperatively in 26.1% (18) of the cases. Cao et al. (35) investigated the clinical and histopathological characteristics of 72 transmasculine individuals with prior testosterone use who underwent hysterectomy. In this group, the leading indication for surgery was pelvic pain in 90% of the cases (n = 65) and gender dysphoria in 26% (n = 19). Eighteen percent (n = 13) of the patients had either endometrial or cervical atrophy, and 22% (n = 16) presented with ovarian or paratubal cysts. BMI, that can be related to aromatization and elevated oestrogen levels, was not associated with the presence of atrophy. Grimstad et al. (37) retrospectively examined clinical and surgical characteristics of transmasculine patients after testosterone use (n = 94). Prior to surgery, 23.1% (n = 12) of the patients complained of breakthrough bleeding and 57.7% (n = 30) of intermittent pelvic pain. Active proliferative endometrium was found in most of the patients (69%, n = 65), atrophic endometrium in 24.5% (n = 23), secretory in 4.3% (n = 4). Endometrial thickness measured by ultrasound was not associated with testosterone use.

Similarly, other studies investigated the presence of breakthrough bleedings in people using testosterone. Grimstad et al. (38) described the ovarian histopathology of transmasculine individuals undergoing oophorectomy as GAC after testosterone use (n = 85). In this population, 49.4% (n = 42) of the subjects were found to have follicular/simple cysts and 4.8% (n = 4) endometriomas. In another study by Grimstad et al. (36), an average of 25% of breakthrough bleeding in patients using testosterone was reported. Patients presenting with persistent bleeding and amenorrheic patients had similar testosterone serum levels (37). However, breakthrough bleedings were more common in patients with longer testosterone use (36). Endometriosis was found to occur significantly more often in patients with breakthrough bleedings (25, 36).

All authors supported the hypothesis that testosterone does not sufficiently suppress endometrial activity for all patients given the high prevalence of proliferative endometrium—up to 69% (37)—and the presence of ovarian activity and ovarian cysts (35, 38) upon testosterone use. Distinctively, longer testosterone use (>12 months) was associated with breakthrough bleedings which were in turn related to endometriosis (25, 35, 36). Only Grimstad et al. (36) examined the estradiol levels in their study population. Estradiol levels were lower in patients without breakthrough bleeding, but not significantly so. However, estradiol levels were significantly higher in the group using testosterone alone compared to those on testosterone and concomitant menstrual suppression therapy.

Different authors advanced the hypothesis that testosterone led to a hyper oestrogenic state due to androgen conversion into oestrogens (25, 36, 37, 39–41). This would explain the persistent ovarian activity, persistent bleedings, and active endometrial tissue. According to this hypothesis, testosterone did not completely suppress endometrial activity, thus endometriosis would likewise remain active (40). It was further discussed that aromatization occurs in adipose tissue but no difference in BMI was found between bleeding and amenorrheic patients. The implication of the changes in endometrial androgen receptor expression in secretory and proliferative phase remained dubious. After having considered different possible causes, the authors concluded that much of the breakthrough bleeding was idiopathic (36).

#### 3.2.2 Relation to individual psychological features

TGD people experience feelings of discomfort around menstruation more often than cisgender women (42, 43). Persistent regular bleedings as well as breakthrough bleedings are often experienced negatively although not by all TGD people (42, 43). The fact that testosterone does not fully suppress the menstrual cycle in all cases can contribute to persistent and even amplified negative feelings around menstruations. After long-term testosterone use (>1 year), breakthrough bleedings are more likely to occur than in the first phase of hormonal therapy (36). This changeover can be particularly challenging for TGD people who have been on a medical transition for a longer time. Aversive feelings around menstruations are associated with increased dysmenorrhea, and, vice versa, painful menstruations impair functional coping with menstruation (22, 42, 44). Particularly painful menstruations, such as in the case of endometriosis, pose an additional burden to this population specifically, as their overall attitude towards the menstrual cycle is frequently negative (42, 43, 45).

### 3.3 Shaping

Shaping (yellow arrow) illustrates how psychological aspects can literally shape someone’s body (Figure 2).

Several behavioural lifestyle factors have been related to the onset and progression of endometriosis (46). Transmasculine people present higher rates for some of these factors than the general population, which include alcohol and substance use, sedentary lifestyle, overweight, and obesity (34, 37, 47). No study directly investigates their confounding effect on endometriosis prevalence among TGD people. Several studies reported psychological traits and comorbidities associated with endometriosis which are more prevalent among TGD individuals compared to cisgender women (37, 48). These are: lower self-esteem and self-efficacy, higher self-criticism, depression, and anxiety (35, 37, 48).

Besides the psychological burden that cisgender women with endometriosis also often carry, gender dysphoria may amplify the psychological distress of TGD patients (24, 41). Physiological processes such as ovulation, breakthrough bleedings, and menstruation are often perceived as related to the female gender. Rydström (42) report how many explain them as “becoming conscious of one’s embodiment as gendered in a way that does not match one’s sense of self” (42, 49). Menarche was perceived as a particularly confronting moment for many transmasculine individuals (42). TGD people who distance themselves from the female gender may experience an augmentation of gender dysphoria when such physiological processes occur (36, 41). This could result in the onset of pain or pain amplification (41). Some authors argue for a better characterization of pain symptoms to distinguish “normal menstruation” from pathological complaints (41). TGD adolescents were particularly susceptible to being disproportionately affected by treatment-resistant endometriosis and presenting with poorer mental health outcomes than TGD adults and their cisgender peers (24, 34, 50). Throughout a person’s life, gender dysphoria was found to affect reproductive choices that were indirectly related to endometriosis. Weak evidence showed that TGD people were more often nulliparous and made less frequent use of combined oral contraceptives than cisgender women (24, 37). This was, for example, because some TGD people discontinued combined oral contraceptives due to chest tenderness and other perceived feminising effects, which thus triggered gender dysphoria (24). The lack of hormonal suppression would favour endometriosis among this study population.

### 3.4 Interaction

Interaction entails the complex processes where psychological and environmental factors interplay.

#### 3.4.1 Psychological risk factors and comorbidities

Individual psychological-environment processes are multileveled and describe the interaction of the social environment with one’s cognitions and behaviours throughout one’s life. Some studies illustrated pathways whereby past events resulted in the onset or progression of a disease later in life (34, 37, 51). For example, several authors investigated the association of a history of trauma, sexual assault, and intimate partner violence with poor mental health outcomes and endometriosis (34, 37, 51). Two studies revealed that these types of traumatic events were more common among TGD people than in cisgender women, thus offering a partial explanation for the gap in endometriosis prevalence between these populations (34, 51). A Portuguese study with a limited sample of five transmasculine and one non-binary individual compared several psychosocial variables of their population compared to cisgender women with endometriosis in the same area (26). The authors report higher levels of pain impact, higher levels of powerlessness and lack of control, lower emotional well-being, lower social support, and worse self-image among their study population compared to the controls (26). The same sample also presented higher mean levels of somatization, depression, and anxiety compared to the general Portuguese population (26). Unfortunately, no study directly investigated the co-founding potential of traumas leaving a gap for adequate quantitative analysis.

#### 3.4.2 Experiencing menstruation

Endometriosis is closely related to menstruation since its typical symptoms occur around the latter (14). The perception and experience of menstruation were found to be culturally and historically dependent (42, 45). The social environment affected menstruation-related individual behaviour (50). Some authors referred to “menstrual etiquette” to indicate the social norms that apply to menstruation in a given culture in time (45). Literature reported experiences from the Global North as well as China, Iran, and Antarctica (42, 45, 50, 52, 53). Recurrent cross-cultural themes were menstruation shame, the need for hiding menstruation, and menstrual taboos. In the retrieved literature menstruation was almost always perceived as related to the female gender. In other words, people menstruating commonly experience bleeding as something that belongs to “women” and “womanhood,” and that it implies physical as well as psychic vulnerability. For example, in China, menstruation was seen as a period of weakened body due to imbalance of yin and yang, and dysmenorrhoea as a sign of poor health (54).

Similarly, across the globe, it was found that menstruation was a challenging moment for menstruating people since they had to deal with menstrual hygiene products and public bathrooms ultimately leading to the reinforcement of traditional binary gender roles (53). Hygiene products were branded exclusively for an all-feminine target audience in terms of both language and design (22, 42, 50). Public bathrooms were—and often still are—usually divided between two exclusive genders. TGD people defined them as unsafe, hostile, and uncomfortable, and they were further reported as spaces of hegemonic patriarchal power, exclusionary of non-cisgender experiences (42, 45, 50). Public bathrooms at the workplace were reported as particularly problematic (45, 50, 55). The difficulties in finding suitable social spaces during menstruation resulted in the adjustment of individual behaviour—e.g. disidentifying from the body—and additional psychological distress (42, 50). The experienced or presumed vulnerability around menstruations was described as contrasting with the desired gender performance of transmasculine individuals (42, 50). In case of endometriosis, the pain symptoms could prevent the person from engaging with their social environment in a manner ideal to them and could expose their gender assigned at birth as well as their emotional and social susceptibility (45, 50). Like menstruation, endometriosis was also narrated as a “women’s thing” in the dominant discourse (56, 57). TGD people struggled to see themselves and their experiences reflected in the narratives of patient groups and online platforms (56). The lack of representation exacerbated their experiences of isolation and negatively impacted their mental health and access to care (57).

Young (53) illustrated how the narrative of “uterine tissue that should not be” exacerbates TGD people’s experience of endometriosis, especially due to their already complex relationship to their bodies. Eder and Roomaney (56) described the feeling of “unhomelikeness” that TGD people with endometriosis experience in relation to their bodies based on focus groups and diaries’ hermeneutic analysis. They investigated how this feeling was triggered through different pathways, including symptoms onset, as well as environmental factors that constitute structural barriers to access adequate healthcare. These feelings of incongruence, disconnect, and “unhomelikeness” led to an amplification of gender dysphoria and consequent pain symptoms as outlined in the shaping process (42).

#### 3.4.3 Identity disruption

The selected literature extensively elaborated on the effects of endometriosis on someone’s identity and specifically on gender identity for cisgender women across different cultures. No study specific to the TGD population was identified. Studies from the Global North, South America, Asia, and Middle East were selected. Many authors referred to the concept of “biographical disruption” for cisgender women living with endometriosis in culturally different settings (48, 52, 58, 59). Cole (58) defined it as the necessary “adjustment to patterns of behaviour; […] how the person with the condition views themselves may change in order to accommodate any effects on their lives and relationships” (p. 173). Cultural gendered expectations relating to relational identities were noted as remarkably challenging (58). Endometriosis was found to be especially disruptive for those aspects of someone’s social life that were seen as related to a female gender role besides the differences in local feminine stereotypes (58). These encompassed expectations in intimate and sexual relationships, such as the ability to enjoy sexual intercourse, but also to do house chores (44, 48, 53, 54). Fertility challenges for endometriosis patients were often narrated as the “failure” to fulfil their gendered expectations as spouses and to become mothers (44, 52, 58). The difficulties in sexual function, fertility, and overall interference of pain symptoms with daily living were found to lead to deep disruptions of someone’s gender identity. Cole (58) reported women feeling “freakish” or “not normal,” “losing the[ir] sense of self” and having a negative body-image (48) evidenced “an extremely negative sense of the patient’s female identity and low self-esteem; “I am only half a woman” (28-years old woman). An adolescent experiencing painful menstruation in rural Colombia was reported to say: “when I menstruate, sometimes I wish to be a man” [trans.] (43). Sexual problems were found to add to the feelings of “femininity” and “unattractiveness” that characterise many ciswomen with endometriosis (59). Impact on the sense of female identity was suggested to decrease self-esteem and self-efficacy, thus contributing to those psychological traits and comorbidities that were found to be associated with endometriosis (48).

#### 3.4.4 Experiences in the healthcare system

Some recent studies focused on the experiences of TGD people across various healthcare systems through qualitative methodologies (27, 56, 57, 60). TGD people face structural barriers to access endometriosis care, some of which overlap with those that cisgender women encounter such as difficulties in being taken seriously by HCPs, while others were unique to this population (56, 57). The authors described TGD-specific barriers as mainly relating to (1) lack of HCP education regarding gender diversity and (2) cisheteronormativity of the healthcare system that was reflected, for example, in gendered language in patients’ health records and communication. Misgendering, discrimination, and cisgender assumptions triggered and intensified feelings of gender dysphoria among TGD people who delayed seeking care for their conditions longer than cisgender women (26, 56, 57). Anxiety regarding physical examinations during gynaecological consults were reported to aggravate this delay (60). The authors suggest that the barriers to access healthcare contribute to further diagnostic delays for this population and potentially lead to underdiagnosis (27, 56, 60). However, how this reflects in the prevalence gap between TGD and cisgender people remains unclear.

### 3.5 Incorporation

The process of incorporation underlies how a living entity embodies certain experiences in their interaction with the environment (28). Incorporation uncovers the effects of environmental exposure over a lifespan in biological changes such as genetic and epigenetic modifications. In the retrieved literature only one study exposed a possible pathway of incorporation, presented with weak evidence due to the nature of the study and highly heterogeneous outcomes under investigation (61). Building on the androgen theory of autism, Ingudomnukul et al. (61) researched women with autism spectrum conditions (ASC) and mothers of children with ASC. Elevated foetal testosterone (FT) levels were correlated with ASC, and this study aimed to demonstrate an association between ASC, “tomboyism,” bisexuality/asexuality, and unusually painful periods in adulthood. Unfortunately, gender identity as such was not investigated. No study was found on the relationships between elevated FT, direct serum testosterone at later points in life, and gender identity/expression among people living with endometriosis.

## 4 Discussion

Transgender and gender diverse people embody specific biomedical, psychological, and social characteristics that result in health needs unique to this community. Endometriosis is a chronic invalidating disease, and it disproportionately affects TGD individuals in comparison to cisgender women.

In this review, we have shown that a developmental embodiment approach was helpful in disentangling the different processes and pathways resulting in the unequally distributed prevalence of endometriosis. However, an exhaustive and comprehensive explanation cannot be provided with the current data. While the challenges related to menstruation for people with gender dysphoria could be more evident, the implications of endometriosis on one’s body perception and gender identity were also found to be profound. Research concerning the barriers in place for TGD people to access adequate and competent endometriosis care is a recent endeavour, but early results suggest these barriers to be of great relevance in the experience of TGD people seeking or receiving endometriosis care. Barriers range from lack of HCP education tosystemic misgendering and discrimination in the healthcare system, and result in an inequitable access to care that disproportionately affects this historically marginalised community.

Endometriosis interferes with physical processes—menstruation, reproduction, and intercourse—that are often associated with the female gender and thus experienced significantly differently by cisgender and TGD people. This interference can lead to biographical disruption and be associated with gender self-perception changes. TGD people have unique ways of relating to their bodies and physiologies throughout their lives, and endometriosis symptoms can amplify feelings of gender dysphoria. It is possible these symptoms cause TDG individuals to feel pressured to seek out gender-affirming care therapies, such as testosterone use and hysterectomies, that have the potential to alleviate them. Conversely, other current endometriosis therapies—such as combined-oral-contraceptives and pelvic floor therapy—are poorly tolerated in this population because they are traditionally associated with the female gender. The scarce utilisation of these first-line treatments could favour endometriosis progression in these individuals. This points out the need for further TGD-centred research and for creating TGD-specific endometriosis guidelines.

### 4.1 Limitations and strengths

The current study presents several limitations. First, the literature available on this topic is still scarce and highly heterogeneous in adopted methodologies. Second, most of the sources were very recent and centred on Global North settings, thus excluding the experiences of TGD people in different geographical areas and limiting the understanding of the historical trajectory underlying this phenomenon. Finally, most studies were conducted within a cisheteronormative framework, and they included only cisgender women or TGD people using hormone GAC.

However, this review also demonstrates several strengths, foremost of which is the emergent and interdisciplinary nature of the topic, allowing for the screening and analysis of a wide range of literature sources. Secondly, to the best of the authors’ knowledge, this is the first time studies on endometriosis conducted within a cisheteronormative framework were analysed from a TGD perspective. This was particularly enriching in explaining the current literature gaps and limitations of contemporary clinical practices. Finally, the authors’ familiarity and connection with the TGD community further fostered a community-based approach and allowed for a more effective understanding of the real lived experiences of TGD people. Whereas the “endometriosis ocean” is deep, the “gender galaxy” is no less vast and this review familiarly navigated small parts of them both (48).

### 4.2 Recommendations for further research

Further research in the field of endometriosis-associated symptoms is imperative, particularly research centering the experiences of those who deviate from cis-hetero and able-bodied norms. Overall, there is a strong need for novel TGD-centred studies, preferably cross-disciplinary, community-based, and trans-led. It is recommended to explore TGD experiences beyond those who make use of medical GAC, as many TGD people do not use any hormone therapy—either because they do not need it, or as a result of barriers to access. Furthermore, quantitative clinical studies are needed on the biological changes induced by testosterone as well as accurate measurements of serum oestradiol in subjects using short-and long-term hormone therapies. Adequacy and acceptability of the current guidelines for endometriosis for the TGD population needs to be tested and challenged to eventually translate research into TGD-competent clinical practice. Additional qualitative studies could support the understanding of the phenomenological aspects of endometriosis among TGD people and the broader implications that the cisheteronormative environment has on bodily characteristics over one’s life. Critical queer, decolonizing, and crip theories could be helpful in supporting the methodologies of qualitative research in this field (22, 62). The current literature linking social factors to psychological features and endometriosis is limited to cross-sectional studies. A longitudinal design would enable an analysis of causality of these correlating factors. Longitudinal studies should also explore how environmental factors are incorporated into individual biomedical characteristics, and why they result in unequal distribution of disease, such as in the case of how foetal testosterone levels can affect one’s behaviour and gender identity later in life. In particular, longitudinal studies investigating the correlation and causation between history of trauma and endometriosis between the TGD and the cisgender population could bring new insights to understand the unequal prevalence of this disease.

## 5 Conclusion

This review explored how TGD people embody endometriosis differently than cisgender women throughout their lives and why this results in a difference in endometriosis prevalence. Overall, TGD people face great barriers to access healthcare, especially reproductive healthcare services, that result in health inequalities between this community and the cisgender population. It is thus essential to reshape healthcare outside cishetero norms to lower these barriers and guarantee health justice for all. More research in this field is urgently needed to overcome the strictly binary narrative of endometriosis as a “women’s disease.” This view is in fact exclusionary of TGD experiences and possibly also damaging to cisgender women’s experience of biographical disruption due to endometriosis. It is essential to start shifting guidelines towards a more inclusive approach to eventually provide more competent care for patients of all genders.

## Data Availability

The original contributions presented in the study are included in the article/supplementary material, further inquiries can be directed to the corresponding author.
